# Student Volunteer Retention and Operational Stability in U.S. Student-Run Free Clinics

**DOI:** 10.1007/s10900-026-01560-3

**Published:** 2026-03-06

**Authors:** Kyle Backston, Yann Chemali, Isha Lodhawala, Amogh Kankanwadi, Jacob Fay, Julie M. Aultman

**Affiliations:** 1 College of Medicine, Northeast Ohio Medical University, Rootstown, OH USA; 2 College of Graduate Studies, Northeast Ohio Medical University, Rootstown, OH USA

**Keywords:** Student-run free clinics, Volunteer retention, Staffing stability, Clinic operations, Continuity of care, Cross-sectional survey

## Abstract

Student-run free clinics (SRFCs) provide essential safety net care for under- or uninsured patients in the United States and are predominantly staffed by student volunteers. Variable volunteer availability and turnover may threaten staffing consistency, clinic operations, and downstream continuity of care. An anonymous, web-based cross-sectional survey was provided to attendees at the 2025 Society of Student-Run Free Clinics annual conference (Chicago, Illinois) using convenience sampling and QR-code recruitment. Two ordinal outcomes, staffing difficulty and operational disruptions, were collapsed into ordered categories. Predictors included clinic characteristics and operational factors. Multivariable ordinal logistic regression models were fit separately for each outcome. Estimates were pooled using Rubin’s rules and reported as odds ratios (ORs) with 95% confidence intervals (CIs). Among 513 conference attendees, 154 responses were obtained (30%), and 152 respondents were identified as having completed the survey. Most clinics used an electronic medical record (83%) and reported high student participation (64% with ≥ 10 volunteers/shift). Staffing difficulty was most often reported as occasional (48%) or rare (32%). Operational disruptions were uncommon (57% never, 34% rare). Higher student volunteer retention beyond 1 year was associated with lower odds of greater staffing difficulty (OR 0.43, 95% CI 0.20–0.94) and lower odds of greater operational disruptions (OR 0.42, 95% CI 0.19–0.93). In SRFCs, student volunteer retention appears to be a central correlate of staffing stability and reduced operational disruption. Operational models that promote longitudinal student engagement may improve clinic reliability and support sustainable delivery of safety net care.

## Introduction

Student-run free clinics (SRFCs) are increasingly vital in providing safety net care to under- or uninsured patients in the United States. SRFCs are typically affiliated with medical schools and mainly staffed by student volunteers. Though student involvement is voluntary, SRFCs are capable of delivering high quality care, meeting or exceeding national metrics [1]. However, students often balance volunteerism alongside a rigorous academic schedule. Students volunteering on an irregular basis may potentiate inconsistencies in clinic operations and patient care [2]. Moreover, high volunteer turnover combined with a limited number of volunteers presents ongoing challenges for care coordination and continuity, with long waitlists for new patients and followup appointments cited as notable concerns [3].

Prior work cites consistency of healthcare services as a recurring theme among SRFCs that prioritize continuity of care [4]. Multiple studies explore strategies to improve healthcare consistency; these include matching patients with student providers longitudinally, incorporating consistent faculty leadership, and the installment of after-care teams [4–6]. While these approaches of improving care continuity are largely successful in SRFCs, they may rely on stable staffing models less affected by volunteer turnover and fluctuations in availability. Because SRFC infrastructures differ significantly by clinic, there is limited data on how these variations in volunteer metrics influence clinic function. Thus, staffing stability remains an underexplored determinant of operational performance in SRFCs. Understanding how student volunteer availability and retention shape clinic operations is essential not only for sustaining SRFCs, but also for informing broader community-based care models that rely on volunteer or trainee workforces. This study aims to identify staffing structures in SRFCs, characterize the role of volunteer availability in clinic staffing consistency, and inform best practices for sustainable clinic operations.

## Methods

### Study Design and Setting

We administered a cross-sectional survey at the 2025 Society of Student-Run Free Clinics (SSRFC) annual conference in Chicago, Illinois. Using convenience sampling, members of the study team approached conference attendees over the duration of the conference and invited them to complete an anonymous web-based survey via a QR code. The survey assessed free clinic characteristics, including geographic region, presence of an electronic medical record system, staffing models, volunteer retention efforts, patient demographics, continuity of care practices, and perceived barriers to operational efficiency. Participation was voluntary, no identifying information was collected, and informed consent was obtained before completion of the survey. This study was approved by the Institutional Review Board at Northeast Ohio Medical University. A waiver of signed informed consent was granted to protect participant confidentiality. The present analysis uses data from the larger cross-sectional survey of conference attendees.

### Study Variables

Survey responses were used to construct 2 ordinal outcomes, staffing difficulty and operational disruptions, each collapsed into ordered categories to improve model stability and address low cell counts. Predictor variables included clinic characteristics and operational factors, including electronic medical record (EMR) use, staffing diversity, student volunteer engagement, and clinic operational strategies. Staffing diversity was transformed into a count of distinct staffing roles present within a clinic (e.g., preclinical students, clinical students, attending physicians, and other support roles), with higher values indicating greater role diversity. Binary predictors were coded as categorical variables with “No” as the reference category. Ordinal predictors were modeled using ordered factors with increasing levels reflecting greater frequency or severity.

### Missing Data

Responses labeled “Not sure/I do not know” were treated as missing data for regression analyses and were not analyzed as a separate categorical response, as they did not reflect an interpretable outcome. Missing data for select covariates were addressed using multiple imputation by chained equations [7]. Multiple imputation allows incomplete observations to contribute information to the analysis by replacing missing values with plausible values drawn from the observed data distribution while accounting for uncertainty. Twenty imputed datasets were generated using appropriate imputation models based on variable type. Outcome variables were not imputed but were included as predictors in the imputation models to improve estimation of missing covariates. Respondents with missing outcome data (n = 2) were excluded in the analytic sample; therefore, 152 total respondents were included in the final pooled analyses.

### Statistical Analysis

The mixed-methods survey included optional open-ended response items, however the present analysis focused exclusively on quantitative data; qualitative analysis is not contained in this manuscript. Categorical variables were summarized using descriptive statistics and presented as frequencies and percentages. Multivariable ordinal logistic regression models were then fit to examine associations between clinic characteristics and each ordinal outcome [8]. Because staffing difficulty and operational disruptions were only moderately correlated (Spearman ρ = 0.37), outcomes were modeled separately to preserve interpretability. Regression coefficients were pooled across imputed datasets using Rubin’s rules, and results are reported as odds ratios (ORs) with 95% confidence intervals (CIs) [9]. Collinearity among predictors was assessed using variance inflation factors (VIFs) derived from the model design matrix. The proportional odds assumption was assessed using the Brant test conducted across multiple imputed datasets [10]. Finally, complete-case analyses were additionally performed as sensitivity checks, with results similar in direction and magnitude to those obtained using multiple imputation. All analyses were performed using R version 2025.05.1 + 513, considering P < 0.05 as significant.

## Results

### Respondent Characteristics and Clinic Infrastructure

**Fig. 1:**
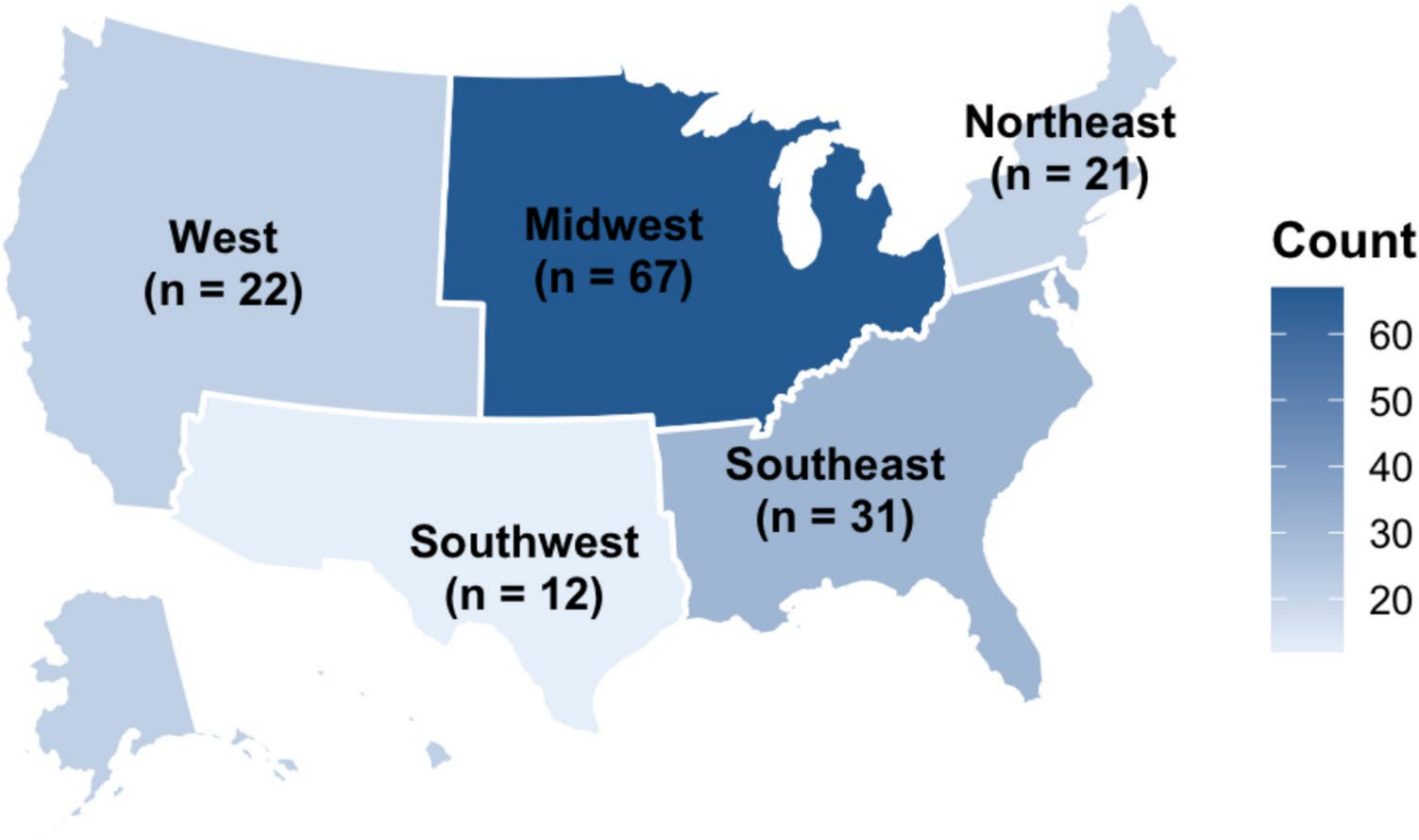
Regional distribution of survey respondents. One respondent located outside the continental United States is not shown. Created using R

Of 513 total present conference attendees, 154 responses were obtained, leading to a response rate of 30%. Descriptive statistics are based on all 154 respondents. Two respondents completed the survey but did not provide responses for the outcome variables; these respondents are included in descriptive statistics where applicable but were excluded from regression analyses. The majority of respondents were from the Midwest, with lower distribution from the West, Southwest, Southeast, and Northeast portions of the continental United States, as shown in Fig. 1.

The majority of surveyed respondents reported established clinical infrastructure, with 83% utilizing an EMR system. A variety of staffing strategies were reported, most frequently involving clinical students (97%) and attending physicians (92%). Most respondents reported treating patients with chronic illness (90%), among others. Student involvement was generally high, with 64% of clinics reporting 10 or more volunteers per shift. Most respondents indicated that student volunteers were likely to be engaged for at least 1 year. However, there was a noticeable loss of student volunteer availability during academic breaks, with 53% reporting moderate decreases, and 31% reporting significant decreases in availability, shown in Table 1.

**Table 1:** Clinic characteristics, patient populations, and student engagement in student-run free clinics (N = 154)

Clinic Domain	Item	Response	n (%)
Clinic infrastructure	Use of electronic medical record (EMR)	Yes	128 (83)
		No	24 (16)
		Not sure	2 (1)
	EMR type^b^	Athena	34 (27)
		EPIC	29 (23)
		Practice Fusion	28 (22)
		Tebra	7 (5)
		Other	7 (5)
		Not specified	23 (18)
Staffing structure^a^	Personnel involved in clinic operations	Preclinical students	131 (85)
		Clinical students	150 (97)
		Attending physicians	142 (92)
		Residents	91 (59)
		Nurses or medical assistants	51 (33)
		Administrative staff	69 (45)
		Social workers	69 (45)
		Pharmacy students	85 (55)
		Nursing students	67 (44)
		Other	43 (28)
Patient populations served ^a^	Populations seen in clinic	Patients with chronic illness	139 (90)
		Patients requiring followup	74 (48)
		Elderly patients	73 (47)
		Patients with mental health or substance use concerns	89 (58)
		Pediatric patients	33 (21)
		Pregnant patients	30 (19)
		Other	18 (12)
Student engagement and participation	Student volunteers per shift	1–3	9 (6)
		4–6	21 (14)
		7–9	25 (16)
		10 +	99 (64)
	Student volunteer likelihood to engage in clinic ≥ 1 year	Always	51 (33)
		Often	81 (53)
		Sometimes	22 (14)
		Rarely	0 (0)
		Never	0 (0)
	Change in student volunteer availability over academic breaks	Significantly decreases	47 (31)
		Moderately decreases	82 (53)
		No significant change	13 (8)
		Moderately increases	3 (2)
		Significantly increases	1 (< 1)
		No operation during academic breaks	8 (5)

^a^Percentages may not sum to 100% due to multiple response options

^b^Percentages for EMR type are calculated among respondents reporting use of an electronic medical record. EMR, electronic medical record

### Staffing Maintenance and Continuity of Care Strategies

Additionally, most respondents reported their clinics implementing both staffing maintenance and care continuity strategies, though a significant proportion of respondents’ clinics either lacked these strategies or were uncertain about their presence. Detailed distributions of staffing maintenance and care continuity strategies are presented in Table 2.

**Table 2:** Staffing sustainability and continuity of care practices in student-run free clinics (N = 154)

Item	Response	n (%)
Staffing maintenance strategies in use	Yes	106 (69)
	No	21 (14)
	Not sure	27 (17)
Care continuity strategies in use	Yes	100 (65)
	No	37 (24)
	Not sure	17 (11)

### Staffing Difficulty and Operational Disruptions

Difficulties in maintaining weekly student volunteer staffing were most commonly reported as occurring occasionally (47%) or rarely (32%), while fewer respondents reported these challenges occurring frequently or very frequently. Operational disruptions due to insufficient student volunteers were less common, with the majority reporting no disruptions in operations (56%) or rare disruptions (33%), as shown in Table 3.

**Table 3:** Staffing challenges and operational impact in student-run free clinics (N = 152)

Item	Response	n (%)
Difficulty maintaining weekly student volunteer staffing^a^	Never	20 (13)
	Rarely	49 (32)
	Occasionally	73 (48)
	Frequently	9 (6)
	Very frequently	1 (< 1)
Operational disruptions due to insufficient student volunteers (past 12 months)^a^	Never	86 (57)
	Rarely	51 (34)
	Occasionally	13 (9)
	Frequently	2 (1)
	Very frequently	0 (0)

^a^Two respondents completed the survey but did not provide responses for the outcome variables; percentages are based on respondents with available outcome responses (N = 152)

### Associations Between Volunteer Retention and Operational Outcomes

In ordinal logistic regression models, respondents reporting higher levels of student volunteer retention beyond 1 year were significantly associated with lower odds of greater difficulty maintaining weekly volunteer staffing (OR: 0.43, 95% CI: 0.20–0.94). Higher student volunteer retention beyond 1 year was also significantly associated with lower odds of greater operational disruptions due to insufficient student volunteers (OR: 0.42, 95% CI: 0.19–0.93). Additionally, although not statistically significant, greater student volunteer availability over academic breaks was directionally consistent in both lower odds of greater staffing difficulty and operational disruptions. Other surveyed factors were not consistently associated with staffing difficulty or operational disruptions after adjustment, as shown in Table 4.

**Table 4:** Multivariable ordinal logistic regression models on staffing difficulty and operational disruptions (N = 152)

Predictor	Staffing difficulty OR (95% CI) ^a^	Operational disruptions OR (95% CI)^a^
EMR use (Yes vs No)	0.98 (0.39–2.50)	0.96 (0.37–2.53)
Staffing structure diversity	0.94 (0.80–1.11)	0.86 (0.72–1.04)
Student volunteers per shift (per category increase)	0.96 (0.37–2.53)	1.25 (0.43–3.67)
Student volunteer retention ≥ 1 year (per category increase)	0.43 (0.20–0.94)*	0.42 (0.19–0.93)*
Student volunteer availability over academic breaks (per category increase)	0.65 (0.29–1.45)	0.84 (0.35–2.00)
Staffing maintenance strategies in use (Yes vs No)	1.64 (0.59–4.60)	0.96 (0.34–2.72)
Continuity of care strategies in use (Yes vs No)	1.06 (0.47–2.41)	0.87 (0.35–2.15)

^a^Odds ratios (ORs) and 95% confidence intervals (CIs) are from multivariable ordinal logistic regression models

^*^Statistically significant (< 0.05). ORs represent the odds of being in a higher (more frequent staffing difficulties or operational disruptions) outcome category versus all lower categories combined. EMR, electronic medical record

## Discussion

### Principal Findings

**Fig. 2:**
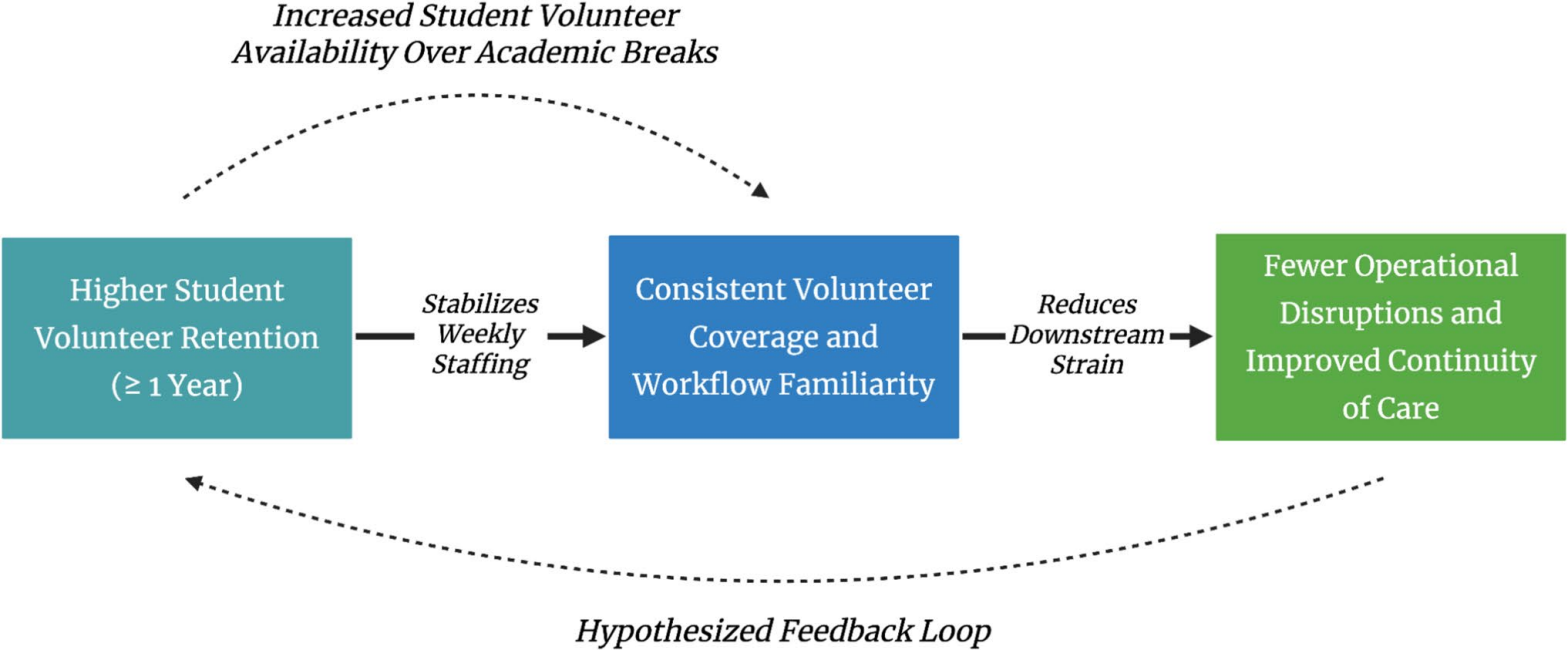
Conceptual pathway linking student volunteer retention to staffing stability and operational outcomes in student-run free clinics. Created in Biorender

The primary goal of this study was to examine how student volunteer availability and retention relate to staffing stability and operational performance in SRFCs. In doing so, we sought to evaluate which staffing-related factors are most strongly associated with operational strain. Our findings suggest that student volunteer retention may play a central role in clinic stability. Specifically, higher levels of retention beyond one year were associated with significantly lower odds of both difficulty maintaining weekly staffing and operational disruptions. The pattern of associations raises the possibility of a linked pathway rather than two isolated outcomes. When volunteer retention is low, clinics may struggle to consistently staff sessions, given the reliance on students to fulfill essential roles. When these challenges persist over time, they may potentially compound into broader operational disruptions. Operational disruptions related to staffing instability may have downstream implications for continuity of care, particularly in SRFCs where sustained student participation supports longitudinal patient engagement. Additionally, student volunteer availability over academic breaks showed a trend toward lower odds of staffing difficulty and operational disruptions, though these associations did not reach significance. Importantly, the direction of these estimates was consistent with the idea that seasonal gaps in availability may represent predictable stress points for SRFC operations. The hypothesized dynamic between student volunteer retention, staffing stability, and operational outcomes is further illustrated in Fig. 2.

In contrast, other examined factors demonstrated non-significant associations with staffing difficulty and operational disruptions (Table 4). While clinic infrastructure development is often emphasized for successful operations, we suggest student volunteer retention may be a prerequisite for infrastructure effectiveness [11–13]. Similarly, the number of student volunteers was not consistently associated with either outcome, suggesting that simply increasing headcount may not be a solution for inconsistent student engagement. Prior SRFC studies also emphasize student engagement and continuity as foundational to clinic sustainability but often discuss this descriptively through leadership turnover and non-aligned student timetables [14, 15]. Our quantitative findings extend this work supporting the hypothesis of clinic success as sequential manifestations of sustained student engagement. In turn, we provide a basis for future research to determine causal relationships between retention, staffing stability, and operational performance.

### Implications for Continuity of Care and Clinic Operations

These findings are particularly relevant when considering periods of predictable disruption in student availability. The widespread decrease in volunteer availability during academic breaks uncovered by our survey may parallel the disruptions observed during the COVID-19 pandemic. Clinics experienced reductions in volunteer capacity not from a lack of interest or commitment, but from external constraints. Academic breaks are full of additional academic or personal obligations, whereas COVID-19 policies restricted in-person participation. Accordingly, we cite a successful hybrid telehealth approach from a reopening SRFC during the COVID-19 pandemic as a potentially translatable solution to the predictable, seasonal declines in volunteer availability [16]. Though, telehealth may also introduce new challenges to successful clinic operations and care continuity [17].

Beyond operational impacts, these findings raise important questions about student engagement with clinic-level staffing and continuity strategies. A substantial portion of survey respondents indicated uncertainty if their clinics had staffing maintenance or care continuity strategies in place (Table 2). This may reflect a potential gap in awareness of systems-level strategies among those involved with SRFCs. Identifiable contributors may include limited communication between clinic leadership and volunteers or an overall reduced exposure to clinic management and systems-level operations. Therefore, while learning outcomes of SRFCs traditionally include clinical and health systems competencies, educational opportunities to engage students in systems-level decision-making may be inconsistently realized in practice [18, 19]. Future research should examine the practical integration of student volunteers in the management of clinic operations.

### Strengths and Limitations

Our study has several strengths. First, it addresses the often underexplored operational aspect of SRFC performance by examining staffing stability and volunteer availability as determinants of clinic performance. By focusing on these parameters, this work contributes to novel insights into how structural challenges affect patient continuity of care in SRFCs. Second, the survey includes clinics from diverse geographic regions and organizational models, enhancing the broader relevance of our findings. Third, the use of multiple imputation to address covariate data that were recoded as analytically missing (“Not sure / I do not know” entries) reduced potential bias while preserving statistical power, allowing these responses to contribute meaningfully to the analysis. Finally, a wide range of clinical characteristics and operational factors were accounted for in the survey, allowing for a comprehensive assessment of factors associated with staffing consistency and clinic function.

Our study has 4 identifiable limitations. First, while all conference attendees were eligible for the survey, some respondents were representing the same clinic, which may overstate the influence of certain clinic-level characteristics found. Second, data points were self-reported and subject to recall bias or misclassification, particularly for questions related to staffing strategies and continuity practices. The study also used self-reported measures of perceived staffing difficulties and operational disruptions rather than objective performance metrics, which may rely on subjective interpretation and variability across respondents. Finally, because clinics differ widely in size, scope of services, and institutional support, there may be unmeasured confounding variables related to local infrastructure or care practices that influenced responses.

## Conclusion

Staffing stability is a critical factor influencing operational performance of SRFCs. This study highlights key associations between volunteer availability and staffing structures in shaping operational disruptions across SRFCs. In doing so, we provide valuable preliminary evidence highlighting staffing stability as an important and underexamined factor in SRFC operations. Given the variability in student volunteer engagement and staffing retention, clinics may benefit from operational models that promote consistency and continuity of care. These findings support the need for further longitudinal studies identifying sustainable staffing practices and may guide future efforts that support the long-term viability of SRFCs as valuable components of community healthcare.

## Data Availability

The datasets generated and/or analyzed during the current study are not publicly available due to institutional and ethical restrictions but are available from the corresponding author on reasonable request.
